# The DIAMOND trial – DIfferent Approaches to MOderate & late preterm Nutrition: Determinants of feed tolerance, body composition and development: protocol of a randomised trial

**DOI:** 10.1186/s12887-018-1195-7

**Published:** 2018-07-07

**Authors:** Frank H. Bloomfield, Jane E. Harding, Michael P. Meyer, Jane M. Alsweiler, Yannan Jiang, Clare R. Wall, Tanith Alexander, on behalf of the DIAMOND Study Group, Tanith Alexander, Jane M. Alsweiler, Sharin Asadi, Friederike Beker, Frank H. Bloomfield, David Cameron-Smith, Clara Y. L. Chong, Caroline A. Crowther, Laura Galante, Jane E. Harding, Yannan Jiang, Michael P. Meyer, Amber Milan, Mariana Muelbert, Justin M. O’Sullivan, Jutta M. van den Boom, Clare R. Wall

**Affiliations:** 1 Liggins Institute, University of Auckland, Private Bag, Auckland, 92019 New Zealand; 2 Newborn Services, Auckland City Hospital, Auckland, New Zealand; 3 The Department of Paediatrics: Child and Youth Health, University of Auckland, Auckland, New Zealand; 4 Department of Statistics, Faculty of Science, University of Auckland, Auckland, New Zealand; 5 Neonatal Unit, Kidz First, Middlemore Hospital, Auckland, New Zealand; 6 Department of Nutrition, Faculty of Medical and Health Sciences, University of Auckland, Auckland, New Zealand

**Keywords:** Preterm, Early nutrition, Growth, Neurodevelopmental outcome, Breastmilk, Taste and smell, Randomised factorial design

## Abstract

**Background:**

Babies born at moderate-late preterm gestations account for > 80% of all preterm births. Although survival is excellent, these babies are at increased risk of adverse neurodevelopmental outcomes. They also are at increased risk of adverse long-term health outcomes, such as cardiovascular disease, obesity and diabetes. There is little evidence guiding optimal nutritional practices in these babies; practice, therefore, varies widely. This factorial design clinical trial will address the role of parenteral nutrition, milk supplementation and exposure of the preterm infant to taste and smell with each feed on time to tolerance of full feeds, adiposity, and neurodevelopment at 2 years.

**Methods/design:**

The DIAMOND trial is a multi-centre, factorial, randomised, controlled clinical trial. A total of 528 babies born between 32^+ 0^ and 35^+ 6^ weeks’ gestation receiving intravenous fluids and whose mothers intend to breastfeed will be randomised to one of eight treatment conditions that include a combination of each of the three interventions: (i) intravenous amino acid solution vs. intravenous dextrose solution until full milk feeds established; (ii) milk supplement vs. exclusive breastmilk, and (iii) taste/smell given or not given before gastric tube feeds. Babies will be excluded if a particular mode of nutrition is clinically indicated or there is a congenital abnormality.

*Primary study outcome:* For parenteral nutrition and milk supplement interventions, body composition at 4 months’ corrected age. For taste/smell intervention, time to full enteral feeds defined as 150 ml.kg^− 1^.day^− 1^ or exclusive breastfeeding. *Secondary outcomes:* Days to full sucking feeds; days in hospital; body composition at discharge; growth to 2 years’ corrected age; development at 2 years’ corrected age; breastfeeding rates.

**Discussion:**

This trial will provide the first direct evidence to inform feeding practices in moderate- to late-preterm infants that will optimise their growth, metabolic and developmental outcomes.

**Trial registration:**

Australian New Zealand Clinical Trials Registry - ACTRN12616001199404. This trial is endorsed by the IMPACT clinical trials network (https://impact.psanz.com.au).

## Background

Of the ~ 11% of babies born preterm each year, > 80% are born moderate- to late-preterm (MLPT) between 32^+ 0^ and 36 completed weeks’ gestation [1]. Although survival of MLPT babies is excellent, these babies constitute a much larger proportion of the health care burden related to prematurity than do extremely preterm babies [1, 2]. Compared to children born at term, MLPT babies have a 36% increased risk for developmental delay or disability at pre-school ages and a 50% increased risk of special education needs at school [3] and account for almost ten times as many children with neurodisability than do extremely preterm babies [4]. MLPT birth also carries an increased risk of adverse long-term health outcomes, including obesity, hypertension and diabetes, even by the 3rd and 4th decades of life [5, 6]. This metabolic risk is substantially related to increased adiposity. Late preterm babies demonstrate an 182% increase in fat mass between birth and term-corrected age, by which time they have ~ 50% greater percentage body fat than term-born controls [7]. This appears to be due to preserved development of fat mass, but impaired accretion of lean mass, indicative of inadequate protein intake between birth and term corrected age [7].

Nutritional practices in early life may impact on later metabolic health through different pathways. A period of relative undernutrition whilst enteral feeds are established may be accompanied by faltering growth which is followed by accelerated growth when nutrition is restored. The postnatal period also represents a critical window for establishing the infant microbiome, which also is associated with later adiposity [8]. More rapid growth in infancy may protect the infant from cognitive impairment but is linked to childhood adiposity, persisting through adulthood [9], suggesting that there may be a trade-off in preterm babies whereby providing enhanced nutrition to prevent postnatal growth faltering results in better brain growth and cognitive outcomes, but accelerates weight gain thus increasing the risk of later metabolic and cardiovascular disease [9].

MLPT babies inevitably experience a delay between birth and the establishment of full enteral feeds due to immature suck/swallow/breathe coordination, immature gut motility, and delayed supply of sufficient breastmilk. Practices around nutritional support for MLPT babies during this period vary widely as there is little high-quality evidence to guide clinical decision making. The usual practice is to provide intravenous fluids while gradually increasing the volumes of milk given by gastric tube until full enteral feeds are tolerated, and then transitioning to sucking feeds as suck/swallow/breathe coordination matures. However, there are many variations within this general approach. There are no data on whether it is better to start supplemental milk early, either donor milk or formula, or to wait until the mother’s breastmilk is available. Whilst waiting for full milk feeds to be tolerated, there are no data on whether the provision of dextrose alone is sufficient, despite the inevitable catabolism and accumulating nitrogen deficit [10], or whether babies should receive parenteral nutrition containing protein. All of these approaches are in use around the world. A study of nutritional support of 33–35 week gestation late-preterm infants in 10 California and Massachusetts hospitals found the rate of intravenous nutrition use varied from 5 to 66% and the rate of discharge with an enriched formula varied from 5 to 71% [11].

Taste and smell also may be important in food tolerance. Even before ingestion of food, taste and smell initiate metabolic processes through secretion of hormones such as insulin and ghrelin [12]. However, the role that these senses play is not usually considered in the care of preterm infants, despite preterm infants having functional taste receptors from 18 weeks’ gestation and flavour perception from around 24 weeks’ gestation [13]. Taste receptors in the mouth relay signals to the brainstem and higher centres, leading to activation of the cephalic phase response and the release of appetite hormones in saliva [14]. These salivary hormones are postulated to play a role in metabolism [14]; indeed, impaired oral nutrient sensing is associated with increased energy intake and a greater body mass index [15]. A pilot trial exposing very preterm infants to the taste and smell of milk before each tube-feed found that infants in the intervention group reached full enteral feeds and tended to have the nasogastric tube removed at an earlier gestational age [16]. These data suggest that the simple intervention of providing taste and smell stimuli before gastric tube feeds may enhance feed tolerance.

Thus, we hypothesise that:

1. Early nutrition supplementation including protein will prevent a protein deficit leading to
  - Body composition at 4 months’ corrected age similar to that of term-born children, and
  - Improved neurodevelopmental outcomes
2. Exposure of MLPT babies to taste and smell before each feed before establishment of full breastfeeds will decrease time to full enteral feeds and full sucking feeds.

### Aims

To investigate the impact of different feeding strategies currently in use on feed tolerance, body composition, and on developmental outcome in MLPT babies.

## Method/design

### Study design

Multi-centre, factorial, randomised, controlled clinical trial.

### Study setting

The neonatal care units in maternity hospitals in Auckland, New Zealand.

### Study population

#### Inclusion criteria

Babies born between 32^+ 0^ and 35^+ 6^ weeks’ gestation, whose mothers intend to breastfeed, who are admitted to the neonatal nursery and require insertion of an intravenous line for clinical reasons.

#### Exclusion criteria

Babies in whom a particular mode of nutrition is clinically indicated or with a congenital abnormality that is likely to affect growth, body composition or neurodevelopmental outcome.

### Interventions and comparators

(i). Parenteral nutrition vs. intravenous dextrose solution;
(ii). Supplemental milk (donor breastmilk if available, else infant formula) vs. only mother’s milk;
(iii). Exposure to taste and smell of milk before every gastric tube feed vs. no exposure (milk administered only via gastric feeding tube).

All babies will receive nutrition according to individual neonatal unit practices. The first two interventions only apply until the baby is established on full enteral feeds with mother’s milk. Babies randomised to receive taste and smell before tube feeds will continue to receive this intervention until the baby is no longer receiving any gastric tube feeds. The goal for all babies enrolled in the study is to transition to full feeds of mother’s breast-milk as soon as possible.

#### Parenteral nutrition

If randomised to receive parenteral nutrition the baby will receive an amino acid solution (according to local hospital practice) intravenously, either by peripheral or central line as deemed clinically appropriate. Administration of lipid is at the discretion of the clinical team, as is the administration of any supplementary fluids, such as 10% dextrose. Babies not randomised to parenteral nutrition will receive dextrose solution with electrolytes as clinically indicated but no protein or lipid. The randomised intravenous fluid will be continued until full enteral feeding is established.

#### Milk supplement

If randomised to receive milk supplement, the baby will receive donor breastmilk or infant formula (according to local practice) while waiting for mother’s breastmilk to meet prescribed fluid amounts. Babies not randomised to receive milk supplement will only receive mother’s breastmilk as available.

#### Taste and smell

If randomised to receive taste and smell, the baby will be exposed to the taste and smell of the milk feed before every gastric tube feed. If the baby is receiving both breastmilk and supplementary formula, the taste and smell will be of breastmilk if available, but if there is insufficient breastmilk, then taste and smell can be of formula. However, if the baby is randomised to not receive supplementary infant formula, then only the taste and smell of breastmilk will be provided with taste given priority if supply is limited.

### Assignment of interventions

#### Allocation sequence generation

Within 24 h of birth, once written consent is obtained, eligible babies will be randomised into one of eight treatment conditions (Table 1) at equal allocation ratio via a secure web-based interface. Randomisation will be stratified by gestation (32^+ 0^ to 33^+ 6^; 34^+ 0^ to 35^+ 6^ weeks), recruitment centre (each centre has different nutrition practices) and sex (this influences growth and body composition), using variable block sizes of 8 or 16. Twins and triplets will be randomised as separate babies.

**Table 1:** Factorial design randomisation table. + means the baby receives this intervention; − means the baby does not

Condition	Parenteral nutrition (i)	Milk supplement (ii)	Taste/smell (iii)
1	**+**	**+**	**+**
2	**+**	**–**	**+**
3	**+**	**+**	**–**
4	**+**	**–**	**–**
5	**–**	**+**	**+**
6	**–**	**–**	**+**
7	**–**	**+**	**–**
8	**–**	**–**	**–**

#### Allocation of concealment mechanism

Randomisation sequence will be computer-generated by the trial statistician and maintained and concealed by an independent database controller until the time of randomisation.

#### Blinding

Due to the nature of the study, it is not possible to blind researchers, clinical staff or families. Researchers involved in the follow-up assessments at 4 and 6 months’ corrected and at 2 years’ corrected age will be blinded to the interventions that the infant received during their admission.

### Study outcomes

#### Primary outcomes

For parenteral nutrition (i) and milk supplement (ii) factors: body composition assessment at 4 months’ corrected age when infant adiposity is predictive of childhood fat mass [17]. For taste/smell factor (iii), time to full enteral feeds, defined as 150 ml.kg^− 1^.day^− 1^ or exclusive breastfeeding if this occurs prior to enteral feeds of 150 ml.kg^− 1^.day^− 1^ being reached.

#### Secondary outcomes

Time to full sucking feeds; number of days in hospital; body composition at discharge; growth: length, weight and head circumference Z-scores and Z-score change from birth to 4 months’ corrected age and at 2 years’ corrected age; developmental assessment at 2 years’ corrected age; breastfeeding rates; nutritional intake from birth to full enteral feeds or until 28 days of age.

### Statistical considerations

#### Sample size

Unlike multi-arm, parallel RCT or comparative experiments, factorial experiments are designed to estimate main effects and their interactions [18]. Each main effect and interaction analysis is, therefore, based upon the total sample size which is chosen to be large enough to detect all primary outcomes [18]; having more factors does not increase total sample size [18]. A total of 480 babies (*n* = 240 per intervention arm) will provide ≥90% power at an overall type 1 error rate of 5% to detect a minimal clinically significant difference in % fat mass at 4 months’ correct age of 3% (lower 95% confidence interval) for parental nutrition and milk supplement interventions, or to detect a reduction in median time to full enteral feeds from 10 to 7 days (hazard ratio 1.43) with the taste/smell intervention. This sample size has assumed a standard deviation of 4% in % fat mass, with Bonferroni corrections to each of the three tests (i.e. alpha per main intervention effect = 0.0167). Allowing for 10% loss to follow-up, we aim to recruit 528 babies (*n* = 66 per randomised condition). The expected effect size is based on an estimated 3% increase in % fat mass in moderate to late preterm infants compared to term infants [7] and an estimated 27% fat mass in term infants at 4 months of age [19]. There are no good data on % fat mass beyond 4 months of age; therefore, this age has been used as the primary outcome.

#### Statistical analyses

Statistical analyses will be performed using SAS version 9.4 (SAS Institute Inc., Cary, NC, USA). The main intervention effects will be evaluated on an intention-to-treat basis. All eligible infants will be analysed according to the assigned condition at randomisation, adjusting for stratification factors and the non-independence of multiple births. Other baseline confounders that are closely associated with the outcomes will be considered in the model if there is evidence of group imbalance by chance (≥ 10%). For the primary outcomes, % fat mass at 4 months’ correct age will be analysed using generalised linear regression with the model-adjusted mean difference. Time to full enteral feeds will be analysed using Cox proportional hazards model with the adjusted hazard ratio. The between group difference will be estimated with 95% confidence interval and *p*-value. An overall type I error rate of 5% will be maintained controlling for multiple comparisons. Secondary outcomes will be evaluated using regression models appropriate to their distributions with similar model adjustment.

Primary analyses will focus on the main effect of each intervention against its comparator, controlling for co-intervention in the same condition. Secondary analyses will test for possible interactions between the main effects. Additional, per protocol analyses will be conducted on those babies without protocol deviations. Missing data will not be imputed on the study outcomes, as the key assumption of missing at random is unlikely to hold in the analysis populations. Sensitivity analyses will be conducted, however, using a multiple imputations method to explore the potential impact of missing data on the primary outcome.

#### Recruitment

Parents of eligible babies will be approached by a member of the research team for recruitment antenatally where appropriate; if antenatal recruitment is not possible than families will be approached after birth upon admission to the neonatal unit. Recruitment will need to occur within 24 h after birth for the baby to be randomised. Formal written consent will be required before babies enter the study. Consented babies who are admitted to the neonatal unit and require an intravenous line will be immediately randomised to one of eight conditions (Table 1). If parents decline consent, nutritional care will be according to the plan of the attending physician.

### Data collection methods

#### Body composition

Body composition will be measured at 4 months’ corrected age when infant adiposity is predictive of childhood fat mass [17]. Measurement using air displacement plethysmography (APD) system PEA POD (COSMED., Concord, CA, USA), will occur as close to discharge as is feasible and at 4 months’ corrected age as the preferred method for determining body composition. Subscapular, triceps, biceps, abdominal, thigh and suprailiac skinfold thickness (mm) will also be measured in triplicate by trained personnel at 4 months’ corrected age using standardised skinfold calipers and the mean value recorded.

#### Anthropometry

Weight, length and head circumference will be measured at birth and every week until discharge and at 4 months’ corrected age.

#### Monitoring of nutritional intake

Total enteral and intravenous intakes will be recorded daily until discharge, or up to 28 days of age, or until baby begins receiving breastfeeds with less than full tube feed top-ups, as the quantity of breastmilk received cannot be quantified. Mean daily protein and energy intakes will be calculated based on actual intakes. Full enteral feeds will be defined as 150 mL.Kg^− 1^.d^− 1^ or exclusive breastfeeding. Energy and protein intakes will be calculated using breastmilk composition for the first week of life (57.1 kcal and 1.9 g protein/100 ml) and for weeks 2–8 (65.6 kcal and 1.27 g protein/100 ml) [20]. For all reporting of neonatal nutrition and growth outcomes, we will use the StRoNNG checklist [21]. Time to full sucking feeds will be defined as until removal of the nasogastric tube for at least 24 h or until discharge home, whichever is the sooner. Any baby discharged home on gastric tube feeds will be excluded from this analysis.

#### Questionnaires

At 4 months’ corrected age mothers will be asked to complete a questionnaire regarding breastfeeding. At 6 months’ corrected age the breastfeeding questionnaire will be administered again over the telephone.

#### Two-year assessments

All surviving children will be assessed formally at two years’ corrected age by trained assessors who will administer the cognitive, motor and language scales of the Bayley Scales of Infant Development, Edition III (BSID III) [22] and undertake a structured assessment of neurodevelopment and growth. The assessment will include a neurological examination to diagnose cerebral palsy (loss of motor function and abnormalities of muscle tone and power). The severity of gross motor problems will be classified using the Gross Motor Function Classification System (GMFCS) [23]. BSID III test scores will be recorded as a standardised normal score [derived from test score - mean/standard deviation (SD)]. Children with severe developmental delay who are unable to complete the assessment will be assigned a standardised score of - 4 SD.

#### Data monitoring and other quality control measures

An independent Data Monitoring Committee (DMC) will be formed to monitor the overal conduct and safety of the interventions during the trial. Aggregate reports of serious adverse events (death, necrotising enterocolitis and any gastrointestinal surgery) and cumulative adverse events (intravenous line extravasation requiring clysis, non-elective removal of central line, confirmed central line-associated blood stream infection and late onset sepsis) will be supplied, in strict confidence, to the DMC by the trial statistician. The Trial Steering Committee will meet within a month of all Data Monitoring Committee meetings to consider their recommendations. An independent Safety Monitoring Committee (SMC) will also be formed. The SMC will review individual reports of serious adverse events. Group allocation will not be revealed to the Safety Monitoring Committee or the investigators. Should the SMC rule that the intervention may have impacted on the adverse outcome, this will be immediately reported to the Steering Committee and if required, to the Chair of the DMC. The Steering Committee will decide on the actions to be taken.

## Discussion

This multi-centre, factorial design clinical trial aims to assess the effects of different feeding strategies in current use for moderate to late preterm infants on body composition, feed tolerance and neurodevelopmental outcome. Until data from large, well-designed randomised trials are available to assess the effects of current feeding strategies on outcomes it is difficult to develop and recommend evidence-based nutrition guidelines. This research has the potential to provide robust evidence to inform feeding practices in moderate- to late-preterm infants that will optimise their growth, development and metabolic outcomes. This will enable us to develop a package of care that will have maximum benefit and, if clinically successful, will not only be cost-effective and economically sustainable but also have the potential to improve long-term health outcomes.

## Abbreviations

ADP: Air displacement plethysmography
BSID III: Bayley Scales of Infant Development Edition III
DIAMOND: Different Approaches to Moderate & late preterm Nutrition: Determinants of feed tolerance body composition and development
DMC: Data Monitoring Committee
GMFCS: Gross Motor Function Classification System
MLPT: Moderate to late preterm
NZ: New Zealand
RCT: Randomised controlled trial
SD: Standard deviation
SMC: Safety monitoring committee
StRoNNG: Standardized reporting of neonatal nutrition and growth outcomes
